# The Effects of Boron Derivatives on Lipid Absorption from the Intestine and on Bile Lipids and Bile Acids of Sprague Dawley Rats

**DOI:** 10.1155/MBD.1995.65

**Published:** 1995

**Authors:** Iris H. Hall, David J. Reynolds, O. T. Wong, A. Sood, B. F. Spielvogel

**Affiliations:** 1 Division of Medicinal Chemistry and Natural Products School of Pharmacy University of North Carolina Chapel Hill North Carolina 27599-7360 USA; 2 Boron Biological, Inc. 620 Hutton Rd. Raleigh North Carolina 27636 USA

## Abstract

N,N-dimethyl-n-octadecylamine borane 1 at 8 mg/kg/day, tetrakis-u-(trimethylamine boranecarboxylato)-bis(trimethyl-carboxyborane)-dicopper(II) 2  at 2.5 mg/kg/day and trimethylamine-carboxyborane 3  at 8 mg/kg/day were evaluated for their effects on bile lipids, bile acids, small intestinal absorption of cholesterol and cholic acid and liver and small intestinal enzyme activities involved in lipid metabolism. The agent administered orally elevated rat bile excretion of lipids, e.g. cholesterol and phospholipids, and compounds 2 and 3 increased the bile flow rate. These agents altered the composition of the bile acids, but there was no significant increase in lithocholic acid which is most lithogenic agent in rats. The three agents did decrease cholesterol absorption from isolated *in situ* intestinal duodenum loops in the presence of drug. Hepatic and small intestinal mucosa enzyme activities, e.g. ATP-dependent citrate lyase, acyl CoA cholesterol acyl transferase, cholsterol-7-α -hydroxylase, *sn* glycerol-3-phosphate acyl transferase, phosphatidylate phosphohydrolase, and lipoprotein lipase, were reduced. However, the boron derivatives 1 and 3 decreased hepatic HMG-CoA reductase activity, the regulatory enzyme for cholesterol synthesis, but the compounds had no effects on small intestinal mucosa HMG-CoA reductase activity. There was no evidence of hepatic cell damage afforded by the drugs based on clinical chemistry values which would induce alterations in bile acid concentrations after treatment of the rat.


### THE EFFECTS OF BORON DERIVATIVES ON LIPID ABSORPTION FROM THE INTESTINE AND ON BILE LIPIDS AND BILE ACIDS OF SPRAGUE DAWLEY RATS

Iris H. Hall, David J. Reynolds, O.T. Wong, A. Sood, and B.F.Spielvogel

Division of Medicinal Chemistry and Natural Products,
School of Pharmacy University of North Carolina,
Chapel Hill, North Carolina 27599-7360, USA and
Boron Biological, Inc., 620 Hutton Rd., Raleigh, North Carolina 27636, USA

ABSTRACT
N,N-dimethyl-n-octadecylamine borane 1 at 8 mg/kg/day, tetrakis-u-(trimethylamine boranecarboxylato)-bis(trimethyl-carboxyborane)-dicopper(II) 2 at 2.5 mg/kg/day and trimethylamine-carboxyborane 3 at 8 mg/kg/day were evaluated for their effects on bile lipids, bile acids, small intestinal absorption of cholesterol and cholic acid and liver and small intestinal enzyme activities involved in lipid metabolism. The agent administered orally elevated rat bile excretion of lipids, e.g. cholesterol and phospholipids, and compounds 2 and 3 increased the bile flow rate. These agents altered the composition of the bile acids, but there was no significant increase in lithocholic acid which is most lithogenic agent in rats. The three agents did decrease cholesterol absorption from isolated *in situ* intestinal duodenum loops in the presence of drug. Hepatic and small intestinal mucosa enzyme activities, e.g. ATP-dependent citrate lyase, acyl CoA cholesterol acyl transferase, cholesterol-7-$\alpha$-hydroxylase, *sn*-glycerol-3-phosphate acyl transferase, phosphatidylate phosphohydrolase, and lipoprotein lipase, were reduced. However, the boron derivatives 1 and 3 decreased hepatic HMG-CoA reductase activity, the regulatory enzyme for cholesterol synthesis, but the compounds had no effects on small intestinal mucosa HMG-CoA reductase activity. There was no evidence of hepatic cell damage afforded by the drugs based on clinical chemistry values which would induce alterations in bile acid concentrations after treatment of the rat.

Keywords: Boron drugs, bile lipids, bile acids, lipid enzymes.

### INTRODUCTION

Previously we have demonstrated that certain boron derivatives are potent hypolipidemic agents in rats at dose ranges of 2.5-20 mg/kg/day. [1-3] These agents demonstrated significant reductions in both rat serum cholesterol and triglyceride levels after 14 days administration. Perhaps more importantly, selected derivatives were able to lower cholesterol content of VLDL and LDL, and elevate HDL cholesterol levels in rats [4]. Preliminary acute toxicity studies have demonstrated that these agents have no deleterious side effects based on organ weights, clinical chemistry levels, hematopoietic parameters and histological examination of major organs of mice and rats [5]. Initial studies on the bile indicated that lipid content was modulated by drug therapy with increases in cholesterol and phospholipid content after 14 days [1-3]. Clinical treatment with hypolipidemic agents have produced cholelithiasis or cholestasis. Therefore, a detailed analysis of rat bile lipid and bile acids after treatment with these agents was addressed along with studies on lipid regulatory enzyme activities and lipid distribution to clarify the characteristics of these agents on bile excretion and intestinal metabolism.

### MATERIALS AND METHODS

N,N-Dimethyl-n-octadecylamine borane 1 was synthesized according to the literature preparation of Hall *et al.* [1]. Tetrakis-u-(trimethylamine-boranecarboxylato)-bis(trimethylamine-carboxyborane)-dicopper(II) 2 was prepared by the method of Hall et al. [2]. Trimethylamine-carboxyborane 3 was synthesized as outlined by Hall *et al.* [3]. Drugs were prepared in 1% carboxymethylcellulose and homogenized. Control animals were maintained on 1% carboxymethylcellulose as a vehicle treated group. All isotopes were purchased from New England Nuclear. Substrates and cofactors were obtained from Sigma Chemical Co., and HPLC column and eluants were obtained from Waters Millipore Co.

Bile Cannulation Studies: Sprague Dawley male rats (~ 300g) were administered compounds 1, 2, or 3 at 8, 2.5, and 8 mg/kg/day, respectively by intubation tube, orally for 14 days. The rats were administered chlorpromazine (25 mg/kg) and anesthetized 30 min later with pentobarbital (22 mg/kg i.p.) [2], a combination which reduces the amount of pentobarbital necessary to maintain anesthesia level over extended periods of time without bronchial spasms. An incision was made just below the rib cage to expose the stomach and the duodenum. Once the bile duct was identified, a loose ligature was placed around it and the duct was nicked. PE-10 plastic tubing was placed in the duct and tied in place. The bile was collected from the control and treated groups over the next 6 hours while the animals were maintained under anesthesia.

Bile Lipid Levels: The flow rate of the bile was calculated for each group as ml/min. Aliquots were extracted for lipids by the Folch *et al.* [6] and Bligh and Dyer [7] methods. Cholesterol [8], triglyceride [Bio-Dynamics/bmc triglyceride kit], neutral lipids [9], and phospholipid content [10] was determined. Protein concentrations were also determined [11]. For HPLC analysis of bile acids content, samples were frozen overnight. After thawing the bile lipids were extracted with EtOH [1:20] and filtered [12]. Aliquots (250 ul) of the bile were added to 10 $\mu$l of the internal standard (testosterone). Then 4.75 ml of hot analytical grade ethanol was added, vortexed, and allowed to evaporate in a boiling water bath. The residues were dissolved in 250 ul of the mobile phase A [acetonitrile-methanol-0.03 M phosphate buffer, pH 3.4 (10:60:30 v/v/v)] and 100 ul was injected onto the HPLC column ($\mu$ Bondapak C18 column) (30 cm x 3.9 mm ID) (Waters) with a guard column (Bondapak C18/Corasi) (Waters) eluted with mobile phase A. The flow rate was 0.5 ml/min (600 psi isobaric flow) with detection at 210 nm. Standard bile acids (Sigma Chemical Co.) were purchased and were treated identical as the bile biological samples.

Absorption from *In Situ* Duodenum Loops: Sprague Dawley male rats were treated orally and anesthesized as indicated above and the duodenum was isolated and nicked. Glass L-shaped cannulae were placed at the proximal end of the duodenum and 20 cm distally down the intestine [13]. The segment was perfused with isotonic PBS Isotope buffer, pH 7.2 until the lumen was clear and all material expelled. Drug solution (0.2 ml) (2.5-8 mg/kg) was placed in the loop with either 1,2-$^3$H- cholesterol (54.8 Ci/mol) or 2,4-$^3$H- cholic acid (25 mCi/mol) Isotope. An aliquot (2uCi) was also placed in the loop. Aliquots (50ul) were periodically removed over the next 210 min and the radioactivity determined using a Packard scintillation counter after correcting for quenching. The disappearance of the isotope from the loop over time was plotted for the control and treated animals.

*In Vivo* $^3$H-Cholesterol or $^3$H Cholic Acid Absorption: Sprague Dawley male rats (~280g) after being treated 14 days with boron derivatives were administered 10uCi of 1,2-$^3$H-cholesterol (54.8 Ci/mol) orally or 2,4-$^3$H-cholic acid (25 mCi/mol) in PBS, pH 7.2, 24 hrs prior to sacrifice [14]. The serum, liver, spleen, small intestine and large intestine mucosa, chyme and a 24 hr fecal specimen were collected. A 10% homogenate of each tissue was prepared in 0.25 M sucrose + 0.001 M EDTA and radioactivity was determined. DPMs were calculated for each total organ.

Hepatic and Small Intestinal Enzymatic Studies: Sprague Dawley male rats (~280 g) were administered drugs 1, 2 or 3, orally for 14 days. On day 15, the animals were sacrificed and the liver and small intestinal mucosa were excised. Homogenates (10%) in 0.25 M sucrose + 0.001 M EDTA, pH 7.2 were prepared of the liver and small intestinal mucosa [15]. The following enzyme assays were determined by literature techniques: ATP dependent citrate lyase [16], acetyl-CoA synthetase [17], HMG-CoA reductase [18, 19], acyl-CoA-cholesterol-acyl transferase [20], neutral-cholesterol-ester-hydrolases [21], cholesterol-7-$\alpha$- hydroxylase [22], acetyl-CoA carboxylase [23], *sn*-glycerol-3-phosphate acyl transferase [24], phosphatidylate phosphohydrolase [25] and lipoprotein lipase [26].

Clinical Chemistry Levels: Blood was collected from the control and treated rats from the abdominal vein after lightly anesthetizing the animal with ether. Sigma chemistry kits were used to analyze SGPT (no. 505), glucose (no. 510) alkaline phosphatase (AP no. 104) LDH (no. 500), and direct bilirubin (no 605). Bile salts and uric acid were determined by the method of Tietz [27]. The animals were killed by carbon dioxide asphyxiation. After all vital signs had ceased, a mid-line incision was made from the lower jaw to the inguinal area. Thymus, spleen, liver and kidney were excised and weighed. Representative tissue samples were fixed in 10% buffered formalin, trimmed and sectioned at 6u in thickness and stained with hematoxylin and eosin. Data displayed in Tables I-VI represent means $\pm$ standard deviations. The Student's "t" test was applied between control groups and the individual drug treatment groups. The analysis of variance (ANOVA) was applied among test drugs and is reported in the text only.

### RESULTS

Treatment with boron derivatives 2 and 3 for 14 days in rats resulted in an increase in the flow rate of the bile whereas compound 1 caused a significant decrease, although the effects of compound 2 were not significant. Compound 1-3 caused increases in cholesterol content in the bile. Compounds 1-3 caused marked increases in bile phospholipids. Only compound 2 caused elevated levels of triglyceride content of the bile (Table I). Neutral lipids in the bile were not affected by any of the drug treatments. Protein content of the bile was elevated by compound 2 and 3. Analysis of the bile acids after treatment with compound 1, 2 and 3 showed that the total bile acids were either increased by drug treatment with 2 but significantly reduced by drug treatment with 1 and 3.(Table II). The ratio of the bile acids were altered in absolute concentration. Compound 1 caused a decrease in taurodeoxycholic acid, taurocholic, taurochenodeoxycholic acid and glycoursodeoxycholic acid levels with an increase in tauroursodeoxycholic acid, glycocholic acids and glycochenodeoxycholic acid content. Compound 2 caused a significant increase in tauroursodeoxycholic acid and glycoursodeoxycholic acid. All other bile acids except taurochenodeoxycholic acid levels were markedly reduced by treatment with compound 2. Compound 3 caused an increase in tauroursodeoxycholic acid, and glycochenodeoxycholic acid content with reduction of all other bile acids except taurocholic acid content.

Table I. The Effects of Boron Derivatives on Bile Lipids of Sprague Dawley Rats Orally for 14 Days

| | Percentage of Control (X ± SD) | | | |
|---|---|---|---|---|
| (*N* = 6) | Control | Compound 1 | Compound 2 | Compound 3 |
| Bile flow rate | 100 ± 4[a] | 50 ± 3* | 119 ± 4 | 151 ± 4* |
| Cholesterol | 100 ± 5[b] | 160 ± 6* | 111.± 8 | 145 ± 4* |
| Triglycerides | 100 ± 7[c] | 87 ± 4 | 238 ± 9* | 106 ± 6 |
| Neutral lipids | 100 ± 8[d] | 104 ± 6 | 101 ± 7 | 98 ± 5 |
| Phospholipids | 100 ± 7[e] | 176 ± 9* | 878 ± 12* | 184 ± 6* |
| Protein | 100 ± 5[f] | 99 ± 4 | 153 ± 7 | 150 ± 6 |

a 0.62 ml/hr  

b 1.18 mg/ml  

c 0.5 mg/ml  

d 1.7 mg/ml  

e 1.75 mg/ml  

f 0.0334 mg/ml  

* $p \leq 0.001$ Student's "t" test

Table II. HPLC Analysis of Bile Acid from Sprague Dawley Rat Treated for 14 Days Orally with Boron Derivatives

| | Percentage of Control (X ± SD) | | | |
|---|---|---|---|---|
| (*N* = 6) | Control | Compound 1 | Compound 2 | Compound 3 |
| Tauroursodeoxycholic Acid[a] | 100[a] ± 3 | 124 ±.3* | 262 ± 6* | 136 ± 5* |
| Glycoursodeoxycholic Acid#[b] | 100[b] ± 6 | 60 ± 6 | 163 ± 5* | 27 ± 3 |
| Taurocholic Acid[c] | 100[c] ± 4 | 0 ± 3* | 0 ± 1* | 97 ± 4 |
| Glycocholic Acid[d] | 100[d] ± 7 | 138 ± 3* | 0 ± 2* | 63 ± 4* |
| Taurodeoxycholic Acid[e] | 100[e] ± 6 | 5 ± 2* | 0 ± 3* | 0 ± 1* |
| Lithocholic Acid[f] | 100[f] ± 6 | 98 ± 4 | 40 ± 4* | 37 ± 3 |
| Taurochenodeoxycholic Acid[g] | 100[g] ± 5 | 10 ± 6 | 100 ± 5 | 0 ± 2* |
| Glycochenodeoxycholic Acid[h] | 100[h] ± 7 | 22 ± 6 | 0 ± 1* | 144 ± 6* |
| Total Bile Acids(mg/ml) | 3.28±0.58 | 10.68±0.73 | 16.93±0.84 | 7.89±53 |

a 2.50 µg/ml  

b 5.61 µg/ml  

c 1.53 µg/ml  

d 0.62 µg/ml  

e 0.50 µg/ml  

f 2.52 µg/ml  

g 0.24 µg/ml  

h 0.09 µg/ml  

\# [glycoursodeoxycholic acid plus tauro-beta muricholic acid]  

* $p \leq 0.001$

The isolated intestinal loop study showed that when the boron derivatives were present at their therapeutic dose, they retarded the absorption of cholesterol from the lumen over 3.5 hr (Table III). Compounds 1 and 3 inhibited the absorption of cholesterol by 50% of that observed in the control. Compound 3 exhibited even greater reduction in absorption at 180 min (ANOVA, $p \leq 0.05$) than compounds 1 and 2. The $^3$H-cholic acid absorption from the loop was not as severely retarded by the three agents over 3.5 hr (Table IV). Compound 1 moderately reduced the amount of cholic acid absorption from the intestinal loop.

Table III. The Effects of Boron Derivatives on $^3$H-Cholesterol Absorption from Isolated Intestinal Loops from Sprague Dawley Rats.

| (N = 6) Time (min) | Percentage Radioactivity in Intestinal Lumen (X ± SD) | | | |
|---|---|---|---|---|
| | Control | Compound 1 | Compound 2 | Compound 3 |
| 0 | 100 ± 5[a] | 100 ± 5 | 100 ± 7 | 100 ± 5 |
| 30 | 70 ± 5* | 76 ± 3 | 75 ± 4 | 90 ± 6 |
| 60 | 43 ± 5* | 69 ± 3* | 58 ± 5* | 57 ± 7 |
| 90 | 35 ± 6* | 64 ± 4* | 41 ± 7* | 74 ± 8* |
| 120 | 29 ± 4* | 64 ± 5* | 36 ± 2* | 96 ± 4* |
| 150 | 22 ± 3* | 60 ± 6* | 44 ± 8* | 97 ± 5* |
| 180 | 21 ± 4* | 85 ± 9* | 48 ± 3* | 111 ± 6 |
| 210 | 10 ± 3* | 88 ± 5* | 58 ± 3* | 111 ± 3 |

[a] 240 dpm/50 ml   * $p \leq 0.001$

Table IV. The Effects of Boron Derivatives on $^3$H-Cholic Acid Absorption from Isolated Intestinal Loops from Sprague Dawley Rats

| (*N* = 6) Time (min) | Percentage Radioactivity in Intestinal Lumen (X ± SD) | | | |
|---|---|---|---|---|
| | Control | Compound 1 | Compound 2 | Compound 3 |
| 0 | 100 ± 4[a] | 100 ± 4[a] | 100 ± 5[a] | 100 ± 5[a] |
| 30 | 68 ± 6* | 73 ± 3* | 66 ± 6* | 70 ± 4* |
| 60 | 45 ± 5* | 39 ± 2* | 46 ± 4* | 34 ± 7* |
| 90 | 24 ± 3* | 28 ± 7* | 30 ± 3* | 13 ± 2* |
| 120 | 10 ± 2* | 29 ± 7* | 33 ± 6* | 15 ± 4* |
| 150 | 5 ± 2* | 35 ± 3* | 26 ± 4* | 13 ± 3* |
| 180 | 2 ± 1* | 36 ± 9* | 10 ± 4* | 6 ± 2* |
| 210 | 1.5 ± 1* | 45 ± 6* | 7 ± 1* | 2 ± 1* |

[a] 460 dpm/50 μl   * $p \leq 0.001$

These data suggest that *in vivo* the boron derivatives interfere with absorption of cholesterol and possibly cholic acid as well as the enterohepatic circulation of these bile acids.

Examination of the hepatic and small intestinal mucosa enzyme activities demonstrated that all three compounds inhibited significantly hepatic ATP-dependent citrate lyase, acetyl-CoA synthetase, acyl-CoA-cholesterol-acyl transferase, and *sn*-glycerol-3-phosphate acyl transferase (Table V). Hepatic phosphatidylate phosphohydrolase activity was reduced by compound 2 significantly and moderately by compound 3. HMG-CoA reductase activity was inhibited in liver cells by compounds 1 and 3. Compound 1-3 caused a reduction of hepatic cholesterol-7-α-hydroxylase activity. Cholesterol-ester-hydrolase activity was elevated by compounds 1 and 2.

Hepatic lipoprotein lipase activity was suppressed by compound 2 and 3. Small intestinal mucosa ATP-dependent citrate lyase activity was suppressed by compounds 1 and 2. HMG-CoA reductase activity was not significantly inhibited by any of the compounds. Acyl-CoA-cholesterol-acyl-transferase activity was reduced in the intestinal mucosa by 1 and 2 but was elevated in the presence of compound 3. Small intestinal mucosa lipoprotein lipase activity was inhibited by compounds 1 and 2.

Table V. The Effects of Boron Derivatives on Sprague Dawley Male Rats Orally for 14 Days on Hepatic and Small Intestinal Mucosa Enzyme Activities

Percentage of Control (X + SD)

| N = 6) | | Liver | | |
|---|---|---|---|---|
| Enzyme Assay | Control | Compound 1 | Compound 2 | Compound 3 |
| ATP-dependent-citrate lyase | 100 ± 4[a] | 7 ± 4* | 25 ± 6* | 19 ± 3* |
| Acetyl-CoA synthetase | 100 ± 5[b] | 4 ± 2* | 33 ± 5* | 22 ± 3* |
| HMG-CoA reductase | 100 ± 6[c] | 50 ± 7* | 128 ± 6* | 55 ± 6* |
| Acyl-CoA-cholesterol-acyl transferase | 100 ± 5[d] | 36 ± 2* | 34 ± 2* | 56 ± 8* |
| Cholesterol-ester-hydrolase | 100 ± 6[e] | 128 ± 7* | 135 ± 7* | 107 ± 7 |
| Cholesterol-7α-hydroxylase | 100 ± 3[f] | 69 ± 7* | 54 ± 4* | 71 ± 7* |
| Acetyl CoA carboxylase | 100 ± 4[g] | 94 ± 7 | 84 ± 6 | 85 ± 8 |
| *sn*-Glycerol-3-phosphate acyl transferase | 100 ± 5[h] | 39 ± 3* | 34 ± 3* | 44 ± 5* |
| Phosphatidylate phosphohydrolase | 100 ± 6[i] | 106 ± 6 | 27 ± 6* | 85 ± 6 |
| Lipoprotein lipase | 100 ± 5[j] | 127 ± 4* | 72 ± 5* | 53 ± 5* |

| (N = 6 ) | | Small Intestinal Mucosa | | |
|---|---|---|---|---|
| Enzyme Assay | Control | Compound 1 | Compound 2 | Compound 3 |
| ATP-dependent-citrate lyase | 100 ± 5[k] | 45 ± 7* | 32 ± 7* | 90 ± 4 |
| Acetyl-CoA synthetase | 100 ± 6[l] | 88 ± 6 | 66 ± 5* | 78 ± 5* |
| HMG-CoA reductase | 100 ± 7[m] | 117 ± 8 | 93 ± 6 | 87 ± 8 |
| Acyl-CoA-cholesterol-acyl transferase | 100 ± 4[n] | 51 ± 8* | 45 ± 4* | 138 ± 9* |
| Cholesterol-ester-hydrolase | 100 ± 6[o] | 237 ± 6* | 63 ± 4* | 87 ± 6 |
| Cholesterol-7α-hydroxylase | 100 ± 4[p] | 65 ± 5* | 18 ± 2* | --- |
| Acetyl CoA carboxylase | 100 ± 6[q] | 99 ± 5 | 83 ± 9 | 101 ± 8 |
| *sn*-Glycerol-3-phosphate acyl transferase | 100 ± 4[r] | 16 ± 4* | 88 ± 6 | 17 ± 4* |
| Phosphatidylate phosphohydrolase | 100 ± 6[s] | 53 ± 6* | 56 ± 5* | 112 ± 6 |
| Lipoprotein lipase | 100 ± 6[t] | 74 ± 4* | 45 ± 6* | 105 ± 7 |

Control values for 60 min/gm wet tissue: a 9.2 mg citrate hydrolyzed; b 10.0 mg acetyl CoA formed; c 103020 dpm; d 86640 dpm; e 22443 dpm; f 289450 dpm; g 43000 dpm; h 87620 dpm; i 11 μg Pi released j 3112 dpm k 9.17 mg citrate hydrolyzed l 5.27 mg acetyl CoA formed; m 113322 dpm; n 64819 dpm; o 23199 dpm; p 259099 dpm; q 54892 dpm; r 73219 dpm; s 114 μg Pi release; t 43128 dpm. * $p \leq 0.001$

Table VI. Effects of Boron Derivatives on Selected Clinical Chemistry Levels of Sprague Dawley Male Rats After 14 Days Orally

| | Percentage of Control (X ± SD) | | | |
|---|---|---|---|---|
| Blood Enzymes | Control | Compound 1 | Compound 2 | Compound 3 |
| SGPT (46.3 m/ml) | 100 ± 6 | 109 ± 5 | 106 ± 4 | 87 ± 4 |
| LDH (102 IU/l) | 100 ± 5 | 86 ± 3 | 99 ± 3 | 80 ± 7 |
| Bilirubin (direct) (0.1 mg/dl) | 100 ± 7 | 95 ± 6 | 104 ± 6 | 99 ± 6 |
| Alkaline phosphatase (15 m/l) | 100 ± 6 | 98 ± 6 | 89 ± 6 | 75 ± 5* |
| Bile Salts | 100 ± 6 | 108 ± 4 | 103 ± 5 | 92 ± 3 |
| Uric Acid (1.5 mg/dl) | 100 ± 5 | 85 ± 3 | 96 ± 4 | 44 ± 5* |
| Glucose (158 mg%) | 100 ± 7 | 88 ± 57 | 92 ± 5 | 101 ± 6 |

* $p \leq 0.001$

When clinical chemistry enzymes and levels were measured in the blood to determine liver function, there were no significant increases in serum bilirubin (direct), glucose, SGPT, LDH, bile salts, or uric acid in any of the sera from treated rats (Table VI). Histological examination of the selected organs after treatment for 14 days with compound 1 demonstrated that the thymus, kidnay and spleen sections were normal. The liver sections showed mild intracytoplasmic vacuolation and capsular reactory hyperplasia with moderate mixtures of acute and chronic inflammatory cells. After treatment with compound 2 the spleen and thymus sections were normal. The liver sections showed increased cytoplasmic vacuolation especically around the central vein. There was one small nidus of acute inflammation of the hepatic capsule. The parenchyma cells of the kidney were normal but there was focal capsulitis and congestion. Compound 3 treatment resulted in no abnormalities of the thymus, kidney, spleen or liver. The control vechicle treated sections of the liver demonstrated scattered collections of mononuclear cells among the hepatic cell cords. The kidney, spleen and thymus sections from the vechicle treated rats were all normal in morphology.

### DISCUSSION

The hypolipidemic agents nafedopin [28] and clofibrate [29] increased the bile flow of rats. Similarly the trimethylamine carboxyborane 3 and dicopper complex 2 increased bile flow in treated rats after 14 days. The addition of boron derivatives into the lumen of the intestine of rats was effective in decreasing the absorption of cholesterol from intestinal loops, suggesting that the drug is effective in blocking the absorption of cholesterol consumed orally in the diet as well as enterohepatic recirculating cholesterol and possibily cholic acids from bile excretion. Previous studies with boron derivatives have shown that orally administered $^3$H-cholesterol to intact treated rats is reduced in the blood after 24 hrs compared to that of control animals [1-3] indicating similar findings after in vivo administration of the boron derivatives. Since a good percentage of the hepatic bile acids are derived from enterohepatic circulation, this mechanism of the drugs would ultimately lower serum cholesterol levels as observed with all three of these agents similar to the clinical hypolipidemic agents colestipol and cholestyramine resins. A direct relationship between the increase in bile acid concentration and bile flow has been noted in rats and monkeys [30]. Certain bile acids are choleretic in rats, for example dehydrocholic acid > chenodeoxycholic acid > cholic acid > taurocholic acid > deoxycholic acid > glycocholic acid. Even though boron derivatives increase the concentration of certain bile acids, there did not appear to be a positive correlation with the effects of boron derivatives on increased levels of known choleretic bile acids and the observed increase in bile flow for compounds 2 and 3. It should be noted that the cannulation technique diverts the bile from the common bile duct. Removing the bile from the intestine could alter the GI mucosa hormonal regulation of pancreatic and gastric mucosa secretion. Thus, these animals may be in a non-physiological state over the 6 hr period. The effects of boron derivatives on GI mucosa hormone secretions were not determined in this study; thus, it is unknown if the drugs altered their levels.

Since hypolipidemic agents, e.g. clofibrate, gemfibrozil and estrogen are known to be lithogenic causing increased incidence of gall stones [31,32]. When used clinically, the effect of boron derivatives as hypolipidemic agents require evaluation in this regard. The boron derivatives altered the concentration of the selected individual bile acids. However, it should be noted that none of the three agents significantly increased lithocholic acid, which in rats has the highest choleretic activity. Tauro- and glycolithocholates are less potent cholestatic agents in rats followed in order by taurodeoxycholate, taurochenodeoxycholate, and taurocholate in rats [33]. The lack of significant increases in any of these three bile acids which would suggest that these boron derivates are not lithogenic at 2.5-8 mg/kg/day for 14 days. No unidentified bile acids were noted in the treated rats in this study. Gall stone formation has been linked with high levels of hepatic HMG-CoA reductase activity and low level levels of cholesterol-7-$\alpha$- hydroxylase activity [29]. In rats it has been observed that the rate of hepatic cholesterol synthesis, the levels of cholesterol esters regulated by acyl-CoA-acyl-transferase activity and the amount of cholesterol absorbed from the intestine play no role in cholesterol secretion into the bile [29]. As observed in the liver enzyme study, the boron derivatives did not demonstrate enhanced hepatic HMG-CoA reductase activity at doses employed. Compounds 2 did increase HMG-CoA reductase activity in the liver 28% but not in the small intestinal mucosa cells. All of the compounds decrease hepatic cholesterol-7-$\alpha$- hydroxylase activity at their respective doses. This is the rate limiting enzyme for the conversion of free hepatic cholesterol to bile acids. In addition, we were able to determine a relatively high concentration of this enzyme in the rat small intestine mucosa cells which was also reduced by drugs 1 and 2 treatment. Acyl-CoA-cholesterol-acyl transferase activity was reduced and cholesterol-ester-hydrolase activity was increased by the three compounds, which should increase the free cholesterol level in the hepatic cell. A good correlation was evident when the drug altered these enzyme activities creating increased level of liver cell free cholesterol and the observed increase of biliary cholesterol levels in treated rats. Another factor in gall stone formation is the insolubility of cholesterol since it is the main component of the stone. Cholesterol is maintained in solution as a mixed micelle consisting of bile acid, cholesterol and phospholipid. Only compounds 2 increased overall bile acid concentrations particularly tarurodeoxycholic acid. Although it should be noted that the bile flow was actually elevated slightly and bile phospholipid concentration was exceedingly high after treatment with this agent. Compound 2 appears to have the greatest potential to be lithogenic but other factors, i.e. the high phospholipid content, may counter its ability to cause gall stones. Compounds 1-3 increased bile phospholipid levels. An increase in bile acid content is related more directly with gall stone formation rather than reductions in phospholipid content in the bile [33]. Estrogen, which is also a hyperlipidemic agent, causes chlolestasis [34] and increases plasma alkaline phosphatase levels indicative of hepatic or other tissue damage. Treatment with the three boron derivatives demonstrated no significant changes in the clinical chemistry values to suggest that the agents damaged the liver cells. Morphological changes were confined to the liver with some changes in the vehicle treated control suggesting some type of mild hepatitis infection of all of the rats and the effects were probably not related to specific drug treatment.

In conclusion, there are significant changes in bile acid composition after treatment with boron derivatives; nevertheless, they do not appear to be lithogenic in nature. The boron derivatives were very effective in increasing biliary cholesterol and phospholipids and thus their elimination form the body since they were not reabsorped. There did not appear to be any morphological lesions or damage to the liver induced by the compounds when administered orally. Thus, the agents did not seem to induce any damage to the hepatic cells which would suggest that they were interfering with their physiological function.

REFERENCES

1. Hall IH, Griffin TS, Docks EL, Brotherton RJ, Futch G. J Pharm Sci 1986; 75: 706-710.
2. Hall IH, Williams Jr. WL, Gilbert CJ, McPhail AT, Spielvogel BF J Pharm Sci 1984; 73: 973-977.
3. Hall IH, Spielvogel BF, Sood A, Ahmed F, Jafri S. J Pharm Sci 1987; 76: 359-365.
4. Hall IH, Spielvogel BF, Griffin TS, Docks EL, Bortherton RJ. Res Comm Chem Path & Pharm 1989: 65: 297-317.
5. Hall IH, Reynolds DJ, Chang JJ, Spielvogel BF, Griffin TS and Docks EL. Arch Pharm 1991; 324: 573-577.
6. Folch J, Lees M, Stanley GHC J Biol Chem 1957; 26: 497-509
7. Bligh EG and Dyer WJ. J Biochem Physiol 1959; 37: 911-917.
8. Ness AT, Pastewka JV, Peacock AC. Clin. Chim. Acta. 1964:10: 229-237.
9. Bragdon JH. J Biol Chem 1951; 190; 513-517.
10. Stewart CP and Hendry EG. Biochem J 1935: 29: 1683-1689.
11. Lowry OH, Rosebrough NJ, Farr AL, and Randall RJ. J Biol Chem 1951; 193: 265-275.
12. Nakayama F and Nakagaki M. J Chrom 1980; 183: 287-293.
13. Doluisio JT, Tan GH, Billups NF, and Diamond L. J Pharm Sci 1969; 58: 1200-1202.
14. Wong OT, Hall IH, Chapman Jr. JM. Pharm Res 1989; 6: 230-234.
15. Chapman Jr. JM, Sowell Sr. JW, Abdalla G, Hall IH, Wong OT. J Pharm Sci 1989; 78: 903-909.

16. Hoffman M, Weiss L, Wieland OH. Anal Biochem 1978; 84: 441-448.
17. Goodridge AG. J Biol Chem 1973; 248: 4318-4327.
18. Haven GT, Krzemian JR, Nguyen TT. Res Commun Chem Pathol Pharmacol 1973; 6: 253-261.
19. Wada F, Hirata K, Sakameto Y. J Biochem Tokyo 1989; 65: 171-175.
20. Balsasubramaniam S, Mitropoulos KA, Venkatesan S. European J Biochem 1978; 90: 377-383.
21. Hall IH, Wong OT, Wyrick SD. Pharm Res 1988; 5: 413-420.
22. Shefer S, Hauser S, Mosbach EH. J Lipid Res 1978; 9: 328-337.
23. Greenspan MD, Lowenstein JM. J Biol Chem 1968; 243: 6273-6280.
24. Lamb RG, Wyrick SD, Piantadosi C. Atherosclerosis 1977; 27: 147-154.
25. Mavis RD, Jacob N, Finkelstein JN, Hall BP. J Lipid Res 1978; 19: 467-477.
26. Chait A, Iverius PH, Brunzell JD. J Clin Invest 1982; 69: 490-493.
27. Tietz NW *Fundamentals of Clinical Chemistry*, Saunders, Philadelphia, 1976; 249-251.
28. Levine WG Drug Met. Dispos. 1974; 2: 178-185.
29. Kutz K, Schulte A, Just C, Linstaedt, and Reiter B. Arch Pharmacol 1979; 308: 171-177.
30. Morris AI, Little JM, Lester R. Digestion 1984; 28: 216-224.
31. Turley SD, Dietschy JM. J Lipid Res 1979; 20: 923-934.
32. Bennion L, Grundy S. New Eng J Med 1978; 299: 1161-1167.
33. Klaossen CD, Watkins III JB. Pharm Res 1984; 36: 1-67.

**Received: September 13, 1994 - Accepted: September 26, 1994 - Received in revised camera-ready format: October 11, 1994**

